# Innate immunity-stroma crosstalk in pancreatic cancer: overcoming spatial and metabolic barriers to immunotherapy

**DOI:** 10.3389/fimmu.2026.1942345

**Published:** 2026-09-30

**Authors:** Yulong Hou, Zhenhua Tan

**Affiliations:** 1 Huzhou Central Hospital, Fifth School of Clinical Medicine of Zhejiang Chinese Medical University, Huzhou, Zhejiang, China; 2 Huzhou Central Hospital, Affiliated Central Hospital of Huzhou University, Huzhou, Zhejiang, China

**Keywords:** cancer-associated fibroblasts (CAFs), immunotherapy resistance, innate immunity, pancreatic ductal adenocarcinoma (PDAC), tumor microenvironment (TME)

## Abstract

Pancreatic ductal adenocarcinoma (PDAC) exhibits profound resistance to current immunotherapies, a challenge largely attributed to its uniquely immunosuppressive and desmoplastic tumor microenvironment (TME). At the heart of this therapeutic recalcitrance lies the dynamic, bidirectional crosstalk between innate immune cells—particularly tumor-associated macrophages (TAMs) and tumor-associated neutrophils (TANs)—and stromal components, notably cancer-associated fibroblasts (CAFs). This review comprehensively delineates how the innate immunity-stroma axis establishes formidable spatial and metabolic barriers that thwart effective antitumor immunity. Spatially, dense collagen matrices, periductal fibroblast organization, and neutrophil extracellular traps (NETs) create physical checkpoints that exclude cytotoxic CD8+ T cells and natural killer (NK) cells from tumor nests. Metabolically, elevated lactate, cholesterol dysregulation, adenosine accumulation, and altered polyamine metabolism synergistically paralyze effector lymphocytes while driving immunosuppressive myeloid polarization. We highlight the key molecular networks orchestrating this pathological crosstalk, including the NF-κB, IL-1β, JAK/STAT3, and TGF-β signaling pathways. Furthermore, we summarize emerging therapeutic strategies designed to dismantle these barriers, encompassing macrophage reprogramming, CAF modulation, and metabolic interventions. Finally, we discuss the multidimensional “3D+R” framework—integrating de-desmoplasia, de-adenosine, *de-novo* antigen presentation, and rational sequencing—as a conceptual roadmap for rationally designed combination therapies to overcome PDAC immunotherapy resistance.

## 1 Introduction

Pancreatic ductal adenocarcinoma (PDAC) remains one of the most lethal malignancies, with a five-year survival rate below 10% and rising incidence worldwide (1, 2). Despite significant advances in cancer immunotherapy, particularly immune checkpoint blockade (ICB) that has revolutionized treatment for multiple solid tumors, PDAC has proven largely refractory to these approaches (3, 4). Clinical trials evaluating anti-PD-1 and anti-CTLA-4 monotherapy have yielded disappointing results, with objective response rates remaining below 5% in unselected patient populations (5, 6). This therapeutic resistance stems from a uniquely hostile tumor microenvironment (TME) characterized by extensive desmoplasia, profound immunosuppression, and metabolic dysregulation that collectively impede effective antitumor immunity (7, 8).

The PDAC TME is distinguished by an abundant fibroinflammatory stroma comprising cancer-associated fibroblasts (CAFs), extracellular matrix (ECM) components, and a diverse array of immune cells (9, 10). This stromal compartment, which can constitute up to 90% of the tumor mass, creates both physical and biochemical barriers that restrict immune cell infiltration and function (11, 12). The dense desmoplastic reaction, driven primarily by activated pancreatic stellate cells (PSCs) and CAFs, generates a collagen-rich matrix that increases interstitial fluid pressure, compresses tumor vasculature, and limits drug penetration (13, 14). Simultaneously, severe hypoxia resulting from aberrant vascularization and high metabolic demand establishes a nutrient-depleted, acidic microenvironment that further suppresses antitumor immune responses (15, 16).

Innate immune cells, including macrophages, neutrophils, myeloid-derived suppressor cells (MDSCs), and natural killer (NK) cells, constitute the predominant immune populations within PDAC tumors (17, 18). Rather than mounting effective antitumor responses, these cells are frequently co-opted by tumor-derived signals to adopt immunosuppressive phenotypes that promote tumor progression and immune evasion (19, 20). Tumor-associated macrophages (TAMs) are particularly abundant and exhibit a spectrum of functional states, with M2-like polarization states dominating in PDAC and correlating with poor prognosis (21, 22). Similarly, tumor-associated neutrophils (TANs) can be reprogrammed toward pro-tumorigenic, immunosuppressive phenotypes that facilitate metastasis and resistance to therapy (23, 24).

The crosstalk between innate immune cells and stromal components represents a critical axis that sustains the immunosuppressive TME and limits immunotherapy efficacy (25, 26). CAFs and TAMs engage in bidirectional signaling through soluble factors, extracellular vesicles, and direct cell-cell contact, establishing positive feedback loops that reinforce each other’s protumor functions (27, 28). This dynamic interaction drives ECM remodeling, metabolic reprogramming, and the recruitment of additional immunosuppressive cells, creating a self-perpetuating cycle of immune exclusion (29, 30). Understanding the molecular mechanisms governing this innate immunity-stroma crosstalk is therefore essential for developing effective combination strategies to overcome immunotherapy resistance in PDAC (31, 32).

## 2 The immunosuppressive landscape of PDAC: innate immune cells and stromal components

The PDAC TME is a complex ecosystem characterized by a dense fibroinflammatory stroma and a predominance of myeloid cells that collectively establish an immunosuppressive milieu (33, 34) (**Figure 1**). Among the most abundant immune populations are TAMs, which can constitute up to 50% of the tumor-infiltrating immune cells and are associated with disease progression, metastasis, and poor survival (35, 36). TAMs in PDAC exhibit remarkable heterogeneity, with single-cell transcriptomic studies identifying multiple subpopulations including SPP1+ lipid-associated macrophages, CCL18+ macrophages, and BST2+ macrophages, each with distinct functional properties (3, 31, 37).

**Figure 1:**
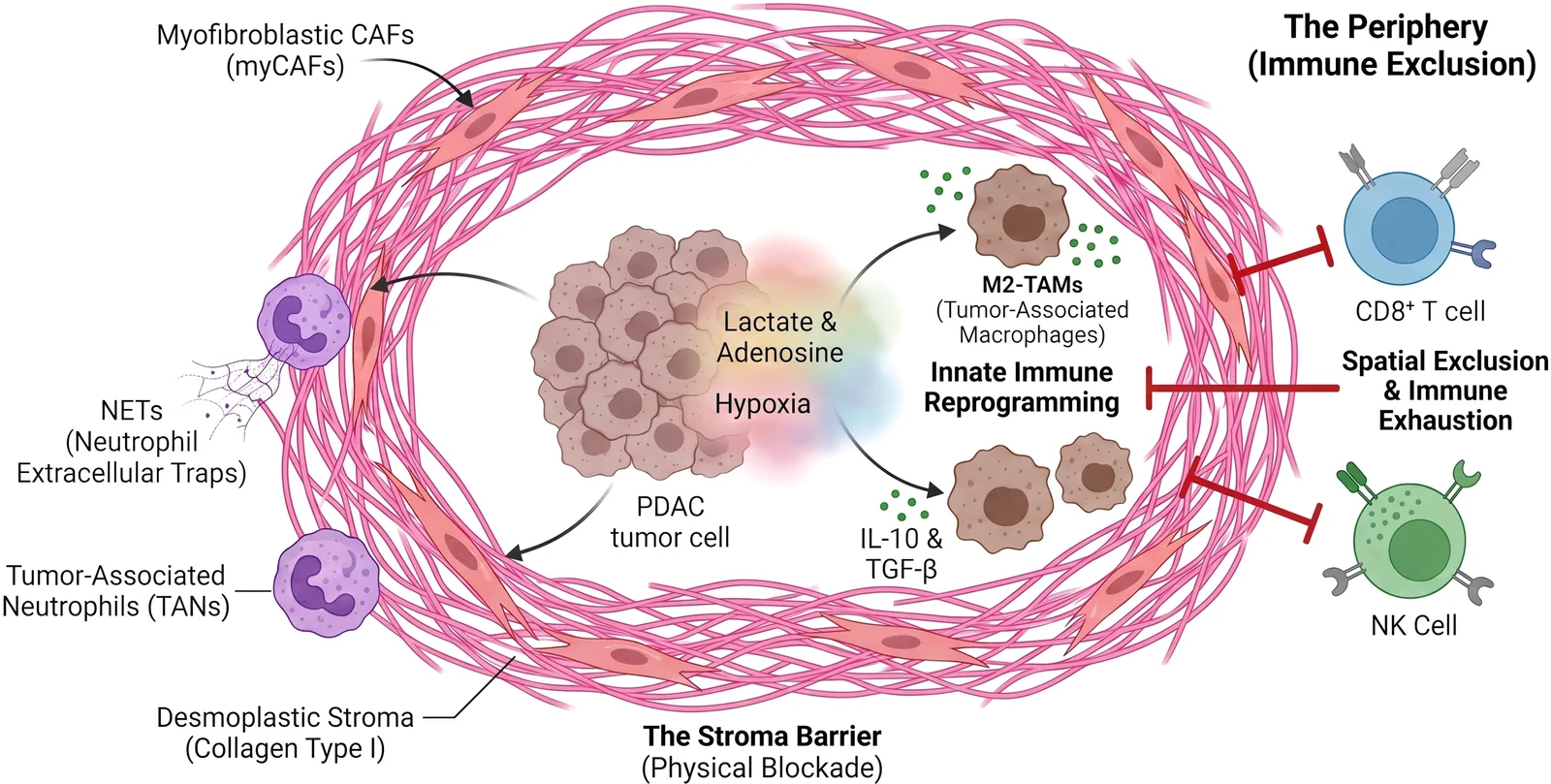
Spatial and metabolic barriers driving immune exclusion in the pancreatic ductal adenocarcinoma (PDAC) microenvironment. PDAC tumor cells create a hypoxic and metabolically hostile milieu enriched in lactate and adenosine. This metabolic dysregulation drives innate immune reprogramming, wherein tumor-associated macrophages (TAMs) are polarized toward an immunosuppressive M2-like phenotype characterized by the secretion of IL-10 and TGF-β. Concurrently, tumor-associated neutrophils (TANs) are recruited and induced to form neutrophil extracellular traps (NETs). Surrounding this core is a dense desmoplastic stroma, primarily composed of type I collagen and myofibroblastic cancer-associated fibroblasts (myCAFs), which functions as a formidable physical blockade. Together, these stroma-derived physical barriers and tumor-driven metabolic/immunological factors orchestrate the spatial exclusion and functional exhaustion of cytotoxic effector cells, including CD8+ T cells and natural killer (NK) cells, thereby profoundly impeding effective antitumor immunity.

The polarization state of TAMs is a critical determinant of their function. While the classical M1/M2 dichotomy provides a useful framework, TAMs in PDAC predominantly adopt an immunosuppressive M2-like phenotype characterized by expression of CD163, CD206, and arginase 1 (ARG1) (35, 38, 39). These macrophages secrete immunosuppressive cytokines such as IL-10 and TGF-β, promote angiogenesis, and suppress cytotoxic T cell activity through multiple mechanisms including arginine depletion and checkpoint ligand expression (35, 38). The recruitment and polarization of TAMs are driven by tumor-derived factors including CCL2, CSF-1, and lactate, establishing a feedforward loop that reinforces immunosuppression (40–42).

Neutrophils and MDSCs represent another major myeloid compartment in PDAC. TANs can exert both pro- and anti-tumorigenic functions depending on their polarization state, but in PDAC they predominantly contribute to immunosuppression (43, 44). TANs promote tumor progression through secretion of CCL5, upregulation of immune checkpoint molecules such as Nectin2, and formation of neutrophil extracellular traps (NETs) that facilitate metastasis and create a physical barrier to immune cell infiltration (29, 45–47). Polymorphonuclear (PMN)-MDSCs, which share phenotypic features with neutrophils, are potent suppressors of T cell function and are recruited to PDAC tumors through CXCR2 signaling (32, 48). The lipid mediator platelet-activating factor (PAF) has been identified as a tumor-derived factor that drives the differentiation of immunosuppressive neutrophils within the TME (49).

NK cells, despite their potent cytotoxic potential, are markedly dysfunctional in PDAC. NK cells are present at very low frequencies within PDAC tumors, and those that do infiltrate exhibit impaired cytotoxicity, reduced expression of activating receptors such as NKG2D and DNAM-1, and defective proliferative capacity (50, 51). The NK cell dysfunction is attributed to multiple factors including TGF-β signaling from CAFs, prostaglandin E2 production, and collagen-mediated signaling that impairs NK cell motility and function (51–53). Importantly, NK cell exclusion from tumor nests is driven by periductal fibroblast organization and collagen-CD44 interactions that create a “stromal checkpoint” limiting immune cell access to malignant epithelial cells (54).

The stromal compartment, particularly CAFs, plays an equally critical role in shaping the immunosuppressive TME. CAFs in PDAC exhibit remarkable heterogeneity, with single-cell analyses identifying multiple subtypes including myofibroblastic CAFs (myCAFs), inflammatory CAFs (iCAFs), antigen-presenting CAFs (apCAFs), and interferon-response CAFs (ifCAFs) (22, 33, 55). MyCAFs are characterized by high expression of α-smooth muscle actin (α-SMA) and collagen production, contributing to the dense desmoplastic stroma, while iCAFs secrete inflammatory cytokines including IL-6, IL-8, and CXCL12 that promote immune suppression (22, 55, 56). A distinct subpopulation of senescent myofibroblastic CAFs (SenCAFs) has been identified that accumulates with PDAC progression and promotes immunosuppression through paracrine signaling (57).

The crosstalk between TAMs and CAFs is bidirectional and self-reinforcing. CAFs promote M2 polarization of macrophages through secretion of CSF-1, IL-6, and sialic acid-containing glycans that engage Siglec receptors on myeloid cells (39, 58). Conversely, TAMs produce factors that activate CAFs and promote their differentiation toward pro-tumorigenic phenotypes (59). This reciprocal interaction establishes a positive feedback loop that sustains stromal desmoplasia and immunosuppression. For example, IL-33 derived from tumor cells activates macrophages to produce CXCL3, which then promotes myCAF differentiation through CXCR2 signaling, creating a TAM-CAF axis that drives metastasis (25). Similarly, TAM-derived IL-1β activates CAFs to produce IL-6, which in turn impairs dendritic cell maturation and T cell activation (60, 61).

The spatial organization of these cell types within the TME is critical for understanding their functional interactions. Imaging mass cytometry and spatial transcriptomics have revealed distinct cellular neighborhoods, including immune-enriched regions, tumor-enriched regions, and a vascular niche where CD44-expressing macrophages interact with endothelial cells (62). Importantly, the juxtatumoral stroma, defined as the stromal compartment immediately adjacent to tumor cells, is characterized by reduced CD8+ T cell infiltration compared to the panstromal compartment, indicating that activated PSCs actively sequester T cells and prevent their access to cancer cells (63). This spatial exclusion is mediated in part by CXCL12 secreted by activated PSCs, which attracts T cells but retains them within the stromal compartment (63).

## 3 Spatial barriers: stromal architecture, immune exclusion, and perineural invasion

The physical architecture of the PDAC TME creates formidable barriers that prevent immune effector cells from accessing tumor cells. The dense desmoplastic stroma, composed of collagen, hyaluronan, and other ECM components, forms a physical barrier that impedes immune cell infiltration and drug penetration (26, 64). This fibrotic stroma is not uniformly distributed but exhibits spatial heterogeneity, with distinct regions of high and low stromal density that correlate with immune cell exclusion (54, 65).

Collagen type I (Col I) is the most abundant ECM component in PDAC, accounting for up to 80% of total ECM collagen (66). Col I deposition exhibits paradoxical effects: heterotrimeric Col I secreted by CAFs initially suppresses tumor growth by recruiting M1 macrophages, while homotrimeric Col I produced by tumor cells promotes immune evasion through CXCL5 secretion and CD8+ T cell reduction (66). The mechanical stiffness of the Col I-rich matrix also activates mechanotransduction pathways in immune cells, influencing their differentiation and function. For example, mechanical force is required for monocyte-to-macrophage differentiation, and the mechanosensitive kinase PYK2 governs this process through PIEZO1 and integrin signaling (7).

The spatial organization of CAFs around tumor ducts is a critical determinant of immune exclusion. Periductal fibroblast density defines lymphocyte exclusion through a CD44-dependent stromal checkpoint (54). In regions with high periductal CAF density, leukocytes are excluded from the epithelial compartment and retained within fibroblast-rich zones, while regions with low CAF density permit leukocyte access to tumor cells (54). This spatial constraint is mediated by collagen-CD44 signaling that anchors immune cells within the stromal compartment, and CD44 blockade enhances NK cell invasion and motility in fibrotic models (51, 54).

The fibrin-rich stroma represents another physical barrier to immune infiltration. In PDAC, *in situ* coagulation creates a thrombotic, crosslinked fibrin-rich tumor stroma (FibTS) that restricts CD8+ T cell and TAM penetration into the tumor parenchyma (65). Tumors with high fibrin content exhibit reduced immune cell infiltration and more aggressive behavior, while inhibition of FibTS formation alters immune cell infiltration patterns and slows tumor growth (65). NETs also contribute to the physical barrier, as they form web-like structures that trap immune cells, promote ECM remodeling, and facilitate metastasis (45–47). NETs are elevated in PDAC and are associated with nearly every stage of tumor progression, from early dissemination to metastatic colonization (46).

Perineural invasion (PNI) represents a unique spatial dimension of PDAC progression that intersects with immune evasion. PNI is a primary route for local tumor dissemination and recurrence, and it also contributes to the severe pain experienced by patients (67). The neural components involved in PNI engage in bidirectional crosstalk with immune and stromal cells, creating a neuro-immune microenvironment that supports tumor progression. Key signaling pathways including CXCL12/CXCR4, TGF-β, and neurotrophin pathways (e.g., NGF/TrkA) coordinate PNI progression while simultaneously shaping the immunosuppressive TME (67). This neural-immune crosstalk suggests that targeting shared regulatory networks could simultaneously disrupt PNI and “heat” the cold TME.

The vascular architecture of PDAC further contributes to immune exclusion. PDAC tumors are characteristically hypovascular, creating a hypoxic environment that alters tumor cell behavior and limits immune cell infiltration (62, 68). The hypoxic TME activates hypoxia-inducible factors (HIFs) in both tumor cells and stromal cells, promoting the expression of immunosuppressive factors including VEGF, IL-6, and CXCL12 (68, 69). Hypoxia-driven fibroblast reprogramming promotes immunosuppressive macrophage phenotypes through IL-6/JAK/STAT3 signaling, while stromal HIF2 regulates immune suppression by recruiting M2 macrophages and regulatory T cells (Tregs) (68, 69).

The spatial distribution of immune cells within PDAC tumors has prognostic significance. Patients with high densities of CD8+ T cells in the juxtatumoral compartment have longer survival times than those with lower densities (63). Similarly, the presence of tertiary lymphoid structures (TLS) is associated with improved prognosis, as these structures facilitate T cell priming and activation (70, 71). Long-term survivors of PDAC exhibit a T cell-inflamed TME with higher densities of CD3+ T cells, CD4+ T cells, and inducible nitric oxide synthase (iNOS)+ leukocytes, and strikingly diminished numbers of CD68+ macrophages, CD163+ M2 macrophages, and FOXP3+ Tregs (71).

## 4 Metabolic barriers: lactate, cholesterol, adenosine, and polyamine-mediated immunosuppression

Metabolic reprogramming is a hallmark of PDAC that profoundly shapes the TME and contributes to immunotherapy resistance. The altered metabolism of cancer cells and stromal cells creates a metabolically hostile environment that suppresses antitumor immune function while supporting tumor progression (72–74). Multiple metabolic pathways converge to establish immunosuppressive conditions, including lactate production, cholesterol metabolism, adenosine accumulation, and polyamine dysregulation.

Lactate is one of the most abundant metabolites in the PDAC TME, produced through aerobic glycolysis (the Warburg effect) driven by oncogenic *KRAS* signaling (13, 72). Elevated lactate levels in PDAC correlate with worse clinical outcomes in immunotherapy and promote an “immune cold” phenotype characterized by abundant pro-tumor macrophages and reduced cytotoxic T cells (13). Lactate exerts its immunosuppressive effects through multiple mechanisms, including histone lactylation that drives transcriptional reprogramming. H3K18la-mediated lactylation promotes the transcriptional activation of ACAT2, which acetylates mitochondrial carrier homolog 2 (MTCH2), stabilizing it and disrupting oxidative phosphorylation to increase lactate production in a positive feedback loop (1). This loop facilitates cholesterol delivery via small extracellular vesicles, polarizing TAMs toward an immunosuppressive M2 phenotype (1).

Non-histone lactylation also plays a critical role in PDAC immunosuppression. Lactate induces K63 lactylation of endosulfine α (ENSA-K63la), which triggers STAT3/CCL2 signaling, leading to TAM recruitment and an immunosuppressive environment (13, 75). Targeting ENSA-K63la or CCL2 enhances the efficacy of ICB therapy in murine and humanized pancreatic tumor models (13, 75). Lactate also promotes macrophage polarization toward the M2d subtype through the lactate/METTL3/OAS3 axis, characterized by elevated secretion of IL-10 and VEGF-A that diminish CD8+ T cell cytotoxicity and promote tumor neoangiogenesis (76).

Cholesterol metabolism is intimately linked to immune regulation in PDAC. The H3K18la/ACAT2 axis drives cholesterol metabolic reprogramming, with ACAT2-mediated cholesterol esterification promoting immunosuppressive TAM polarization (1). Apolipoprotein E (ApoE), elevated in TAMs and peripheral blood monocytes of PDAC patients, induces tumor cell expression of CXCL1 and CXCL5 through LDL receptor and NF-κB signaling, suppressing CD8+ T cell infiltration (21). High serum ApoE levels correlate with poor patient survival, and ApoE-deficient mice exhibit reduced tumor growth with increased CD8+ T cell infiltration (21). Metabolic landscape analyses have identified a potential crosstalk whereby TAMs provide cholesterol to cancer cells, with TAMs upregulating cholesterol export while cancer cells upregulate lipid/cholesterol receptor import (77).

The adenosine pathway represents another critical metabolic barrier in PDAC. The CD39-CD73-adenosine axis generates pathologically elevated adenosine concentrations that systematically suppress anti-tumor immunity through four G-protein-coupled receptors (A1, A2A, A2B, A3) (78). A2A and A2B receptors mediate the predominant immunosuppressive effects through cAMP-PKA signaling, inhibiting CD8+ T cell and NK cell cytotoxicity while enhancing Tregs, MDSCs, and M2-like TAMs (78). CAFs and tumor cells contribute to adenosine production and respond to its signaling, establishing self-reinforcing immunosuppressive networks (78). Spatial analysis using mass spectrometry imaging has revealed that extracellular adenosine distribution is heterogeneous in PDAC tumors, with high concentrations in peri-necrotic, hypoxic regions associated with rich myeloid infiltration (79). Blocking adenosine formation and function using a combination of anti-CD73 antibody and an A2A receptor inhibitor slows tumor growth, reduces metastatic burden, and improves the efficacy of cytotoxic agents and immunotherapy (79).

Polyamine metabolism is dysregulated in PDAC and contributes to immunosuppression. Spermine levels are significantly elevated in the plasma of PDAC patients and correlate with poor prognosis (17). Mechanistically, Serpinb9 interacts with spermine synthase (SMS), impeding its lysosome-mediated degradation and augmenting spermine production and secretion (17). The accumulation of spermine in the TME alters the metabolic landscape of immune cells, promoting CD8+ T cell dysfunction and pro-tumor polarization of macrophages (17, 80). PDAC is uniquely dependent on glutamine-derived ornithine for *de novo* polyamine synthesis, orchestrated by the *KRAS-MYC* axis, and targeting polyamine metabolism represents a promising strategy to improve immunotherapy outcomes (80).

The metabolic landscape of PDAC exhibits cell type-specific alterations that collectively contribute to immunosuppression. Single-cell metabolic analyses have revealed that cancer cells enhance biosynthetic programs while immune populations, including granulocytes, B cells, and CD8+ T cells, downregulate mitochondrial programs through cell type-specific mechanisms (77). Macrophage metabolism is particularly altered, with TAMs exhibiting elevated glycolysis and lipid metabolism (72, 77, 81). Macrophage-specific deletion of glucose transporter 1 (GLUT1) significantly reduces tumor burden, accompanied by increased NK and CD8+ T cell activity and suppression of the NLRP3-IL1β inflammasome axis (81). TAMs drive glycolysis in PDAC cells through the IL-8/STAT3/GLUT3 signaling pathway, creating a reciprocal metabolic interaction that fuels tumor progression (40).

## 5 Mechanistic crosstalk: signaling pathways driving innate-stroma interactions

The bidirectional crosstalk between innate immune cells and stromal components in PDAC is governed by a network of signaling pathways that collectively establish and maintain the immunosuppressive TME. Understanding these pathways is essential for identifying therapeutic targets that can disrupt the innate-stroma axis and restore antitumor immunity.

The NF-κB pathway serves as a central hub connecting inflammation, immune suppression, and stromal activation in PDAC. Constitutive and induced activation of NF-κB transcription factors drives inflammation in PDAC, promoting the expression of immunosuppressive cytokines, chemokines, and checkpoint ligands (82, 83). IL-1 receptor-associated kinase 4 (IRAK4) drives NF-κB activity in PDAC cells, and its inhibition abrogates the expression of multiple immunosuppressive factors, checkpoint ligands, and hyaluronan synthase 2 (82). Conditional deletion or pharmacologic inhibition of IRAK4 decreases tumor desmoplasia and increases the abundance and activity of infiltrative CD4+ and CD8+ T cells, setting the stage for successful combination with checkpoint immunotherapy (82).

The IL-1β signaling axis is a critical mediator of innate-stroma crosstalk. Tumor cell-derived IL-1β promotes the activation and secretory phenotype of quiescent PSCs and establishes an immunosuppressive milieu mediated by M2 macrophages, MDSCs, and regulatory B cells (61). IL-1β production by tumor cells is driven by microbial-dependent activation of TLR4 signaling and subsequent engagement of the NLRP3 inflammasome (61). NLRP3 signaling in macrophages drives the expansion of immune-suppressive macrophages and promotes the differentiation of CD4+ T cells into tumor-promoting Th2, Th17, and Treg populations while suppressing Th1 polarization and cytotoxic CD8+ T cell activation (84). The IL-33-ST2 pathway also contributes to innate-stroma crosstalk, as IL-33-activated macrophages produce CXCL3 through MYC signaling, promoting myCAF differentiation and PDAC metastasis (25).

The JAK/STAT3 pathway is a major convergence point for multiple immunosuppressive signals in PDAC. STAT3 signaling in CAFs promotes an immunosuppressive microenvironment by increasing CXCL1 secretion, which drives M2 macrophage polarization (56). STAT3 activation in myeloid cells is also critical for MDSC differentiation and function. PSCs promote MDSC differentiation through IL-6-mediated STAT3 phosphorylation, and STAT3 inhibition abrogates PSC-mediated MDSC differentiation (85). The STAT3/CCL2 axis is activated by ENSA-K63 lactylation, promoting TAM recruitment and immunosuppression (13, 75). In chimeric antigen receptor (CAR)-T cells, CXCR4 co-expression impairs STAT3 signaling, reducing inflammatory factor release and suppressing CXCL12 secretion from CAFs, thereby decreasing MDSC migration (86).

The TGF-β signaling pathway plays a dual role in PDAC, with both tumor-suppressive and tumor-promoting functions. TGF-β depletion by AVID200/BMS-986416 (TGF-β-TRAP) potentiates the efficacy of anti-PD-1 therapy in PDAC models, significantly reducing liver metastases (87). TGF-β-TRAP modulates CAF heterogeneity and suppresses neutrophil degranulation and CD4+ T cell response to neutrophil degranulation (87). TGF-β signaling also drives myCAF differentiation, and TGF-β-driven myCAFs are strong NK cell suppressors, in contrast to iCAFs (52). The dual-specific anti-PD-L1/TGF-β antibody BiTP synergizes with chemotherapy in neoadjuvant settings, reprogramming the TME by increasing penetration and function of T cells, NK cells, and dendritic cells while decreasing immunosuppressive cells (88).

The CXCR2 signaling axis is a major chemotactic pathway for myeloid cell recruitment to PDAC tumors. CXCR2 ligands, including CXCL1, CXCL5, and CXCL8, are among the most abundant soluble factors released by PDAC cells, and their expression is at least partially dependent on mutant *KRAS* and NF-κB signaling (48). CXCR2+ MDSCs are abundant in PDAC tumors and suppress effector cell function (32, 48). Cell-autonomous *Cxcl1* expression in *KRAS-TP53* co-altered PDAC mediates spatial T cell restriction through interactions with CXCR2+ neutrophilic MDSCs (32). Neutrophil-derived TNF is a central regulator of this immunologic rewiring, instigating feedforward CXCL1 overproduction from tumor cells and CAFs, T cell dysfunction, and inflammatory CAF polarization via transmembrane TNF-TNFR2 interactions (32, 89). CXCR2 blockade overcomes NETosis-mediated resistance to MEK inhibition, blocking TAN infiltration to hypoxic/necrotic areas and improving antitumor immunity (90).

The cGAS-STING pathway represents an innate immune sensing mechanism that can either promote or suppress antitumor immunity depending on context. STING agonists promote an ifCAF phenotype *in vivo* and *in vitro*, suppressing tumor cell invasiveness and inducing an antitumor phenotype in TANs (22). cGAS and STING co-expressing PDAC cases show favorable survival with many cytotoxic CD8+ T cell infiltrations, while cGAS-STING signaling-defective PDAC cases exhibit reduced immune infiltration (91). However, STING signaling can also be co-opted by tumors to promote immunosuppression. Nanoparticle delivery of STING and TLR4 agonists combined with senescence-inducing agents promotes T cell control of PDAC through type I interferon-mediated innate and adaptive immune responses (92).

The PI3K-AKT pathway is a critical regulator of macrophage function in PDAC. Macrophage PI3Kγ drives immunosuppressive transcriptional programming that inhibits adaptive immune responses and promotes tumor cell invasion and desmoplasia (93). Blockade of PI3Kγ in PDAC-bearing mice reprograms TAMs to stimulate CD8+ T cell-mediated tumor suppression and inhibit tumor cell invasion, metastasis, and desmoplasia (93). Granulocyte-macrophage colony-stimulating factor (GM-CSF), released from cancer cells in a mutant *KRAS*-dependent manner, dysregulates TAM gene expression and metabolism through PI3K-AKT pathway signaling (41).

## 6 Therapeutic strategies to overcome immunotherapy resistance: targeting innate-stroma crosstalk

The growing understanding of innate-stroma crosstalk in PDAC has led to the development of multiple therapeutic strategies aimed at overcoming spatial and metabolic barriers to immunotherapy. These approaches target different components of the innate-stroma axis, including macrophage reprogramming, CAF modulation, metabolic intervention, and combination therapies (**Table 1**).

**Table 1:** Key therapeutic strategies targeting the innate-stroma axis in PDAC.

Target/strategy	Agent/drug class	Stage of evidence	Mechanism of action	Clinical trial examples
Macrophage Reprogramming	CSF1R Inhibitors (e.g., Cabiralizumab)	Clinical (Phase I/II) & Preclinical	Depletes M2-like TAMs; Reprograms TME when combined with ICB	NCT03336216 (with Nivolumab)
	CD40 Agonists (e.g., Sotigalimab)	Clinical (Phase II)	Licenses DCs for T cell priming; Shifts TAMs to M1-like state	NCT03214250 (with chemo ± Nivolumab)
	CCR2/CCR5 Inhibitors (e.g., BMS-813160)	Clinical (Phase I/II)	Blocks monocyte recruitment to the TME	NCT03184870 (with chemo ± Nivolumab)
Stromal Normalization	FAP-CAR Macrophages/T cells	Preclinical	Targets and degrades FAP+ myCAFs and collagen	N/A (Preclinical *in vivo*)
	Senolytics (Dasatinib + Quercetin)	Preclinical	Eliminates senescent CAFs to relieve immune suppression	N/A (Preclinical *in vivo*)
Metabolic Intervention	CD73/CD39 Inhibitors (e.g., Oleclumab)	Clinical (Phase I/II)	Blocks adenosine generation; relieves T/NK cell suppression	NCT03611556 (with Durvalumab)
	SLC4A4 Targeting	Preclinical	Mitigates tumor acidosis; reverses macrophage immunosuppression	N/A (Preclinical murine models)

Macrophage-targeted therapies have shown promise in preclinical models and early clinical trials. CSF1R blockade reprograms TAMs, enhancing antigen presentation and productive antitumor T cell responses (42). However, CSF1R blockade also upregulates T cell checkpoint molecules including PD-L1 and CTLA-4, providing a rationale for combining CSF1R inhibitors with checkpoint blockade (42). Combined CSF1R and checkpoint inhibitor therapy potently elicits tumor regressions in preclinical murine PDAC models (42). Similarly, in preclinical *in vivo* studies, CSF1R inhibition using AZD7507 causes shrinkage of established PDAC tumors and increases mouse survival, with malignant cell proliferation diminishing and increased T cell immune response (36).

CD40 agonists represent another approach to reprogram the myeloid compartment. CD40 activation licenses dendritic cells to prime antitumor T cell responses, and agonistic CD40 antibodies have shown early promising activity in ongoing human PDAC clinical trials (60, 94). Combination of CD40 agonists with chemotherapy and checkpoint blockade is being investigated, with the goal of converting immunologically cold tumors into hot ones (94).

Targeting the CCL2/CCR2 axis disrupts monocyte recruitment to PDAC tumors. CCR2 is primarily expressed on macrophages in PDAC, and CCR2 knockout significantly prolongs survival in autochthonous PDAC models, with a shift in macrophage composition toward iNOS+ pro-inflammatory macrophages and decreased tumor microinvasion (95). CCR2 blockade suppresses PDAC progression through interaction with macrophages in the TME, and combination of CCR2/CCR5 inhibition with radiation therapy and anti-PD-1 enhances effector T cell infiltration (96). The dual CCR2/CCR5 inhibitor BMS-813160 is currently being evaluated in Phase I/II human clinical trials for PDAC (97).

CAF-directed therapies aim to normalize the stromal compartment rather than eliminate it entirely, as complete stromal ablation can paradoxically accelerate tumor progression. Targeting CAF senescence has emerged as a promising strategy, as depletion of senescent stromal cells relieves immune suppression by macrophages, delays tumor progression, and increases responsiveness to chemotherapy (57). The senolytic drug combination dasatinib plus quercetin is being explored in preclinical models and awaits further clinical validation (57).

FAP-targeted approaches represent another CAF-directed strategy. Chimeric antigen receptor (CAR) macrophages targeting fibroblast activation protein-α (FAP) on activated CAFs enhance chemotherapy and immunotherapy by removing the fibrosis barrier (15). In preclinical assessments, FAP-CAR-M treatment decreases CAF markers, collagen volume fraction, and Col I secretion, improving drug and immune cell penetration (15). Similarly, FAP-CAR-T cells are being developed to target the stromal compartment (15).

Metabolic interventions target the metabolic vulnerabilities of the PDAC TME. Lactate-targeting strategies include inhibition of lactate production through glycolysis or mutant KRAS inhibition, which reshapes the TME and increases sensitivity to ICB therapy (13). Targeting the bicarbonate transporter SLC4A4 mitigates tumor acidosis, improves T cell-mediated immune response, and breaches macrophage-mediated immunosuppression (98). In preclinical models, SLC4A4 targeting in combination with ICB overcomes immunotherapy resistance and prolongs survival (98).

Adenosine pathway inhibition represents another metabolic intervention. The CD39-CD73-adenosine axis can be targeted at multiple levels, including CD73 inhibitors, CD39 antagonists, and adenosine receptor antagonists (78). Multiple CD73 inhibitors are currently under evaluation in human Phase I/II PDAC trials, and combination of adenosine pathway inhibition with checkpoint blockade shows enhanced efficacy in both preclinical models and early clinical settings (78, 79). However, CD73’s non-enzymatic functions promote chemoresistance independently of adenosine production, suggesting that multi-targeted approaches may be necessary (78).

Polyamine metabolism is an emerging therapeutic target. Disrupting the Serpinb9-SMS interaction with small peptides significantly enhances the efficacy of ICB therapy (17). Polyamine-targeted therapy, including biosynthesis inhibitors, arginine deprivation agents, and polyamine analogues, represents a promising strategy to reverse immunosuppression and enhance checkpoint inhibitor efficacy (80).

Combination therapies that simultaneously target multiple components of the innate-stroma axis are likely to be most effective. The combination of STING agonists with senescence-inducing agents promotes T cell control of PDAC through type I interferon-mediated innate and adaptive immune responses (92). Similarly, combining radiotherapy with immunotherapy can transform immunologically cold tumors into hot ones by inducing immunogenic cell death, enhancing antigen presentation, and modulating cytokine and chemokine profiles (99, 100).

The combination of neoadjuvant chemoradiotherapy with immunotherapy has shown sex-specific effects, with women deriving greater benefit due to enhanced antitumor immunity and reduced protumoral M2 macrophage infiltration (101). This finding highlights the importance of considering sex disparities in treatment response and personalizing immunotherapy approaches (101).

Despite robust efficacy in preclinical murine models, translating these stroma- and innate-targeted strategies to the clinic has proven exceptionally challenging. A primary reason for clinical failure is the immense plasticity and redundancy of the PDAC TME. For instance, while targeting a single axis like CSF1R effectively depletes TAMs in mice, it often triggers compensatory upregulation of alternative immunosuppressive pathways, such as T cell checkpoint molecules (e.g., PD-L1 and CTLA-4), rapidly negating therapeutic benefits (42). Furthermore, early clinical attempts aimed at complete stromal ablation failed spectacularly. For example, the inhibition of Sonic Hedgehog (Shh) signaling or non-specific depletion of α-SMA+ myofibroblasts, which effectively reduced desmoplasia in preclinical models, paradoxically induced immunosuppression and accelerated tumor progression with reduced survival (102, 103). This clinical failure taught the field a critical lesson regarding CAF heterogeneity: the stroma contains both tumor-promoting (myCAFs, iCAFs) and tumor-restraining subpopulations (22, 55). This underscores that rationally guided “stromal normalization, “ rather than broad stromal depletion, is required. Finally, translational failures are exacerbated by the limitations of current preclinical models. Commonly used subcutaneous or fast-growing syngeneic models often fail to fully capture the chronic desmoplasia, spatial complexity, and prolonged immune exhaustion characteristic of human PDAC (104). Bridging this translational gap requires a shift from single-agent interventions to rationally designed, multi-targeted approaches evaluated in more physiologically accurate, slowly evolving genetically engineered mouse models (GEMMs) or patient-derived explants that account for patient-intrinsic factors.

## 7 Conclusion and future perspectives

The innate immunity-stroma crosstalk in PDAC represents a formidable barrier to effective immunotherapy, but also offers multiple therapeutic vulnerabilities. The spatial exclusion of immune effector cells from tumor nests, driven by periductal fibroblast organization, dense ECM deposition, and aberrant vascular architecture, prevents T cells and NK cells from accessing cancer cells (26, 54, 63). The metabolic reprogramming of the TME, characterized by elevated lactate, dysregulated cholesterol metabolism, adenosine accumulation, and polyamine dysregulation, creates a metabolically hostile environment that suppresses antitumor immune function while promoting immune tolerance (13, 72, 78, 80).

The bidirectional crosstalk between TAMs and CAFs is central to establishing and maintaining these barriers. TAMs and CAFs engage in reciprocal activation through multiple signaling pathways including IL-1β, TGF-β, STAT3, and NF-κB, creating self-reinforcing loops that sustain stromal desmoplasia and immunosuppression (56, 59, 61, 82). Understanding these interactions at the molecular level has revealed multiple therapeutic targets, including CSF1R, CCR2, CXCR2, CD40, and various metabolic enzymes (42, 48, 94, 97).

Despite significant progress, several challenges remain. The heterogeneity of CAF and TAM populations, their plasticity, and their context-dependent functions complicate therapeutic targeting (33, 55). Complete ablation of stromal components can be detrimental, as some CAF populations exert tumor-suppressive functions (22). Similarly, metabolic interventions must be carefully designed to avoid toxicity and to account for the metabolic flexibility of cancer cells (78).

Emerging technologies are providing new insights into the spatial organization and functional interactions of innate and stromal cells in PDAC. Spatial transcriptomics and imaging mass cytometry have revealed distinct cellular neighborhoods and ligand-receptor interactions that govern immune cell positioning and function (62, 105, 106). Single-cell RNA sequencing has identified novel cell subtypes and differentiation trajectories that were previously unrecognized (3, 14, 55, 107). These technologies, combined with advances in metabolomics and proteomics, are generating comprehensive multi-omic datasets that can guide the development of precision immunotherapies (74).

Future research should focus on several key areas. First, the development of predictive biomarkers that can identify patients most likely to benefit from specific immunotherapy combinations is essential for clinical translation (108). Second, the optimization of treatment sequencing and combination strategies, including the timing of radiotherapy, chemotherapy, and immunotherapy, will be critical for maximizing therapeutic efficacy (99, 100). Third, the integration of metabolic interventions with immunotherapy requires careful consideration of the metabolic dependencies of both cancer cells and immune cells (73, 74).

To overcome the multifaceted resistance mechanisms in PDAC, the concept of the “3D+R” framework—de-desmoplasia, de-adenosine, *de-novo* antigen, and rational sequencing—provides a multidimensional conceptual roadmap (108) (**Figure 2**). The validity of this framework is increasingly supported by recent synergistic therapeutic successes. For instance, relieving spatial exclusion through de-desmoplasia is a prerequisite for immune infiltration. Preclinical evidence demonstrates that modifying the dense extracellular matrix, such as by targeting focal adhesion kinase (FAK) or employing FAP-CAR macrophages (15), effectively breaches the fibrotic barricade. Crucially, while these stroma-normalizing agents alone are insufficient, they can successfully render previously cold PDAC tumors highly responsive to subsequent checkpoint immunotherapy (16). Simultaneously, overcoming the metabolically hostile TME represents the de-adenosine pillar. Given that the CD39-CD73-adenosine axis profoundly paralyzes infiltrating effector cells, combining adenosine pathway inhibitors with anti-PD-1 therapy significantly unleashes CD8+ T cell and NK cell cytotoxicity while restraining M2-TAMs (78, 79).

**Figure 2:**
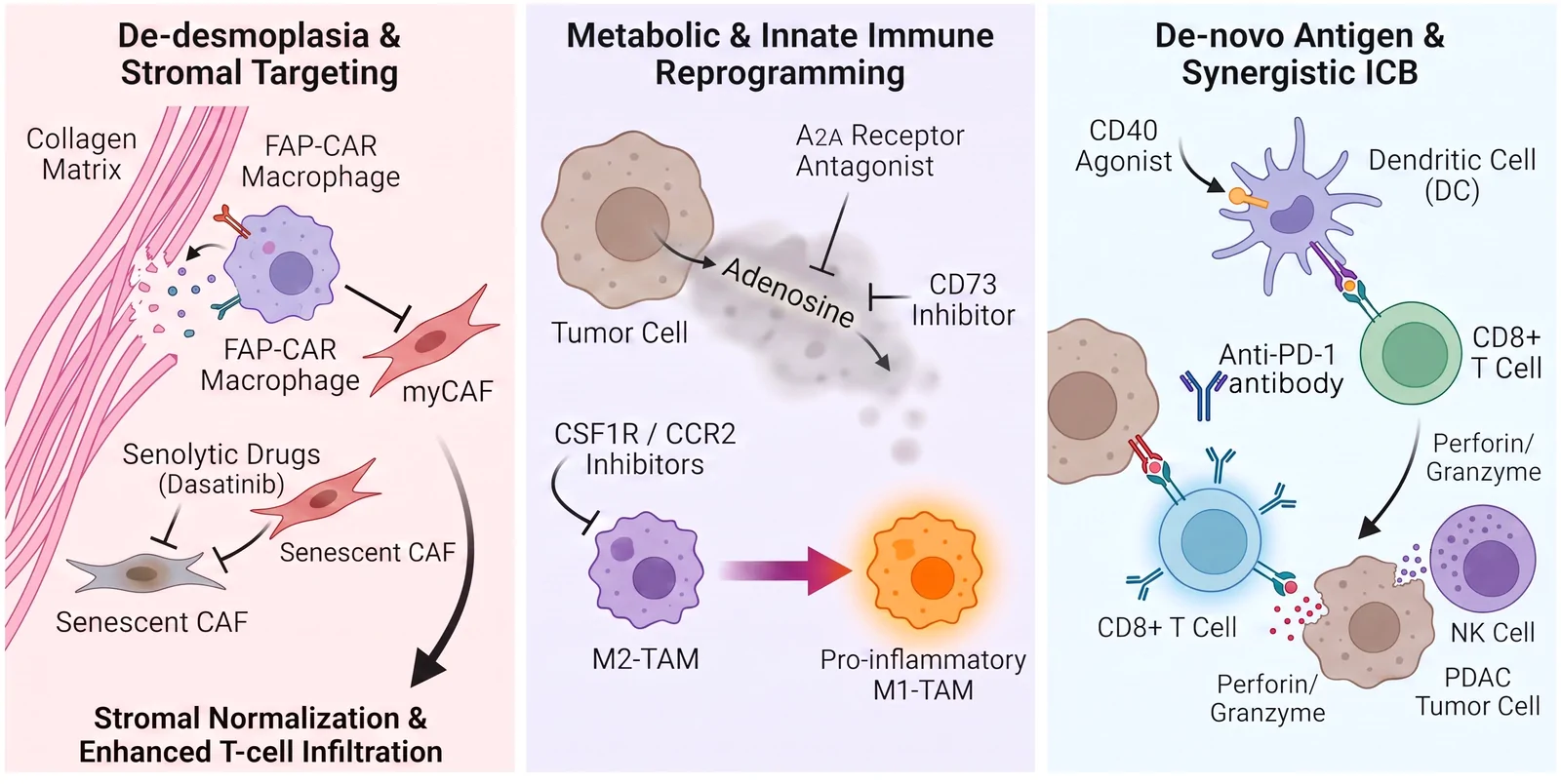
The multidimensional “3D+R” framework for overcoming immunotherapy resistance in PDAC. (Left) De-desmoplasia and stromal targeting: The physical stromal barrier is normalized using FAP-CAR macrophages to target myofibroblastic cancer-associated fibroblasts (myCAFs) and degrade the collagen matrix, alongside senolytic drugs (e.g., dasatinib) to eliminate senescent CAFs, thereby enhancing T-cell infiltration. (Middle) Metabolic and innate immune reprogramming: The immunosuppressive adenosine signaling axis (“de-adenosine”) is blocked via CD73 inhibitors and A2A receptor antagonists. Simultaneously, immunosuppressive M2-like tumor-associated macrophages (TAMs) are reprogrammed into a pro-inflammatory M1-like state using CSF1R or CCR2 inhibitors. (Right) *De-novo* antigen presentation and synergistic ICB: CD40 agonists license dendritic cells (DCs) to effectively prime T cells (“*de-novo* antigen”). This functions synergistically with anti-PD-1 antibodies (immune checkpoint blockade, ICB) to restore and sustain the cytotoxic activity—mediated by perforin and granzyme release—of CD8+ T cells and natural killer (NK) cells against PDAC tumor cells. (Not depicted spatially) The framework is completed by the ‘+R’ component (Rational sequencing), emphasizing that the optimal temporal arrangement of these interventions (e.g., stromal degradation logically preceding effector cell infusion, or neoadjuvant chemoradiotherapy preceding immunotherapy) is critical for maximizing synergistic anti-tumor efficacy.

Even with physical and metabolic barriers dismantled, anti-tumor immunity requires robust antigen presentation, underscoring the need for *de-novo* antigen priming. The use of CD40 agonists to license dendritic cells for *de novo* T cell priming exemplifies this approach (94), and ongoing trials show that combining CD40 agonists with stroma-modulating chemotherapy profoundly shifts the myeloid compartment and primes new T cell clones (60). Finally, the rational sequencing component highlights that the temporal arrangement of these interventions is as critical as the agents themselves. Administering FAP-CAR macrophages to degrade the collagen matrix must logically precede the infusion of effector T cells (15). Clinically, the rational sequencing of neoadjuvant chemoradiotherapy prior to immunotherapy induces immunogenic cell death, modulates cytokine profiles, and heats the TME, thereby maximizing the efficacy of subsequent interventions (100, 101). By integrating these physical, metabolic, and immunological interventions in a rationally sequenced manner, it may be possible to convert PDAC from an immune-desert tumor to an immune-reactive one.

In conclusion, the innate immunity-stroma crosstalk in PDAC creates a unique ecosystem that supports tumor progression and resists immunotherapy. The spatial and metabolic barriers established by this crosstalk must be overcome for immunotherapy to be effective. While challenges remain, the growing understanding of the molecular mechanisms governing these interactions, combined with advances in therapeutic modalities including CAR-T cells, bispecific antibodies, and metabolic inhibitors, offers hope for improving outcomes in this devastating disease. Continued investigation of the dynamic interactions between innate immune cells, stromal components, and tumor cells will be essential for developing effective therapeutic strategies that can finally bring the benefits of immunotherapy to patients with pancreatic cancer.
